# Towards developing a comprehensive treatment schedule for patients with cerebral palsy: factors influencing patient's adherence to physiotherapy treatment

**DOI:** 10.4314/ahs.v22i2.66

**Published:** 2022-06

**Authors:** Chigozie Uchenwoke Ikenna, Chidimma Ofodum Mirian, Chinonso Nwachukwu Paul, Christiana Osuoha Onyekachi, Kenechukwu Okonkwo Kingsley, Lynda Anih Chidera

**Affiliations:** Department of Medical Rehabilitation, Faculty of Health Sciences and Technology, College of Medicine, University of Nigeria, Enugu Campus, Enugu State, Nigeria. www.unn.edu.ng

**Keywords:** Cerebral palsy, Treatment schedule, Adherence, Physiotherapy, Nigeria

## Abstract

**Purpose:**

This study is aimed to identify factors influencing patient's adherence to treatment in a bid to characterize the extent to which these factors are considered while developing a treatment schedule for patients with cerebral palsy in Nigeria

**Methods:**

Descriptive cross-sectional study of physiotherapists involved in the care of patients with cerebral palsy. Factors influencing treatment adherence were assessed using a pre-tested, self-administered questionnaire. Participants were sampled from physiotherapists working at University of Nigeria Teaching Hospital, Enugu State, Nigeria. The data were analyzed using descriptive statistics of percentage and frequencies.

**Results:**

A total of fifty three (31 males and 22 females) physiotherapists completed and returned the questionnaire. Participants (84.9%) agreed that patients occasionally forget to meet up with their appointment days; with majority of them agreeing that distance to the clinic and economic factor (cost of treatment and transportation) influence patient's adherence to treatment. Presence/absence of a caregiver and relationship between patients and their physiotherapist are also important factors influencing patient's treatment schedules.

**Conclusion:**

Distance to the clinic when compared to other (economic, patient-therapist relationship) factors is the major barrier to patient's adherence to treatment and therefore should be considered while developing treatment schedules for patients with cerebral palsy

## Introduction

Cerebral Palsy (CP), a group of movement and posture disorders attributed to non-progressive disturbance that occurs in a developing infant/fetal brain^1^; is the most common motor disability in childhood ranging from 1.5 to more than 4 per 1000 live births^2^, ^3^, ^4^, ^5^ in occurrence. CP displays variation in presentation, etiology, evolution, severity, comorbidities and outcomes,^6^ thus emphasizing the need for individualized medical and rehabilitative interventions which has the potential to help children with CP engage in meaningful life activities.

CP management requires a multi-disciplinary approach in which physiotherapy plays a vital role in^7^. The goal of physiotherapy is to help patients with cerebral palsy achieve their potential of physical independence and fitness levels within their community while improving their quality of life and those of their caregivers who have a major role to play in the process^7^. By developing a comprehensive treatment plan and schedule according to the specific needs of patients, a physiotherapist addresses limitation of mobility and achieve optimal results in patients with cerebral palsy. Treatment schedule, a vital part of cerebral palsy intervention constitutes a standard list of appropriate interventions to be used, how it is administered and how often (such as daily or weekly) the therapy would be required^8^. Treatment schedule is greatly associated to the prognosis and effectiveness of physiotherapy management^9^ underscoring the need for such schedules to be strictly followed as planned. Failure to keep scheduled clinical appointments has negative implications and is being reported to disrupt clinic workflow, stimulate negative clinician attitudes, hinder the delivery of optimal quality care and cause premature discontinuation of clinical service^10^. In other words, a well-planned treatment schedule can be marred by poor adherence, thus resulting to a poor treatment outcome, recovery time and quality of life.

Since children with cerebral palsy and other developmental disabilities require long term rehabilitation programs and complex management regimes^11^ in which they are largely expected to be adherent to; a crucial step in improving adherence to physiotherapy treatment of such patients is to ensure that the treatment schedules are consciously developed in such a way to encourage patient's adherence. Identifying the factors which could influence patient's adherence is pertinent in informing treatment schedules decision. Essentially, factors such as severity of the condition, distance to the clinic, economic status of patient, age of patient^12^ have been considered to play a key role in determining adherence and subsequently; the treatment schedule for patients with cerebral palsy. However, there is no evidence suggesting that these factors are well appreciated and considered by physiotherapists while scheduling patients' appointments and schedules especially for children with cerebral palsy. The lack of a comprehensive guideline for developing treatment schedules for patients with cerebral palsy which features these variables have implications in the level of patient's compliance to treatment and hinders uniformity in patient's management. Therefore, as a crucial step towards developing a comprehensive treatment schedules for patients with cerebral palsy, this study aims to identify factors influencing patient's adherence to treatment in a bid to characterize the extent to which these factors are considered while developing a treatment schedule for patients with cerebral palsy in Nigeria

## Methods

### Study Site

This study was conducted in University of Nigeria Teaching Hospital, Ituku-Ozalla, Nigeria. An average of 12–15 patients with cerebral palsy are managed daily in the hospital.

### Study Design

A descriptive cross-sectional survey design was employed.

### Study Participants

A minimum sample size of fifty-six (56) participants (physiotherapists) was achieved from a population size of sixty physiotherapists working in University of Nigeria Teaching Hospital Ituku/Ozalla. In order to achieve this sample size, all physiotherapists working in the above-named hospital were recruited as it acts a major referral centre in the Eastern Nigeria and individuals who were not willing to sign the informed consent form were excluded from the study.

### Data Collection

Physiotherapists working at the University of Nigeria Teaching Hospital were approached for written consent after the purpose of the study was adequately explained. A pre-tested self-administered questionnaire was utilized to determine factors influencing the treatment schedule of patients with cerebral palsy. The questionnaire is divided into two sections: the first section contains the sociodemographic characteristics of participants while the second part constitutes specific questions addressing factors that can influence adherence. The questionnaire was prepared after a robust literature review with face validity of the questionnaire assessed by the authors to ensure that it answered its set objectives. Pre-testing of the questionnaire was done among physiotherapists from a nearby state government facility who were not included in this study. Sixty copies of the questionnaire were printed and distributed to the participants and they were given five days to answer the questions after which the questionnaires were retrieved for data analysis.

### Ethical Considerations

The ethical approval was sought and obtained from the Medical Research and Ethics Committee of the University of Nigeria Teaching Hospital, Ituku-Ozalla, Nigeria. Participation was entirely voluntary, and authors ensured confidentiality as codes were used as personal identifiers rather than participants' names.

### Method of data analysis

Data was analyzed using Statistical Package for Social Sciences (SPSS) software version 21. Software. Description statistics of percentage and frequency were used to summarize participant's demographic characteristics and questions pertaining to factors influencing treatment schedules of patients with cerebral palsy.

## Results

Sixty copies of the questionnaire were distributed to physiotherapists in University of Nigeria Teaching Hospital. However, a total of fifty-three (53) physiotherapist responded to the survey yielding a response rate of 88%. The highest number of responses were found among male physiotherapists, aged between 21–30 years and have been in practice for 6–10 years. Details are found in Table 1.

**Table 1:** Socio-demographic Characteristics of Participants

Variables		Frequency	Cumulative frequency	Percentage %
	21–25	5	5	9.4
**Age (Years)**	26–30	17	22	32.1
	31–35	9	31	17.0
	36–40	10	41	18.9
	41–45	6	47	11.3
	46–50	5	52	9.4
	> 50	1	53	1.9
**Gender**	Male	31	31	58.5
	Female	22	53	41.5
**Rank**	Physiotherapist	21	21	39.6
	Senior PT	20	41	37.7
	Principal PT	7	48	13.2
	Chief PT	5	53	9.4
**Years of practice**	1–5	20	21	39.6
	6–10	21	41	37.7
	11–15	1	42	1.9
	16–20	4	46	7.5
	21–25	5	51	9.4
	26–30	1	52	1.9
	>30	1	53	1.9

PT- Physiotherapist

### Factors influencing patient's adherence

The perceived factors influencing the treatment schedules of patients with cerebral palsy are illustrated in Table 2. The factors were broadly grouped into patient's compliance, economic, distance and patient-therapist relationship factors. Participants stated their opinions on the various sub-questions of the broad questions using a 5-point Likert scale.

**Table 2:** Factors influencing patient's adherence

Variables		Strongly agree	Agree	No Opinion	Disagree	Strongly Disagree
Patient's adherence	Does your patient attend appointments fixed by physiotherapist?	56.6%(30)	34.0%(18)	1.9%(1)	7.5%(4)	0
	Does your patient attend weekly appointments?	52.8%(28)	47.2%(25)	0	0	0
	Does your patient attend the appointments more than twice a month?	73.6%(39)	26.4%(14)	0	0	0
	Do your patients occasionally, forget to come on the treatment schedule days?	26.4%(14)	58.5%(31)	5.7%(3)	9.4%(5)	0
Distance related factors	Does the distance of the clinic affect your patient coming for treatment	35.8%(19)	52.8%(28)	3.8%(2)	7.5%(4)	0
	Does the absence of caregiver sometimes prevent your patient from coming to clinic	58.5%(31)	39.6%(21)	0	1.9%(1)	0
Economic factor	Does transportation cost influence your patient from coming for treatment?	45.3%(24)	54.7%(29)	0	0	0
	Does cost of treatment influence your patient from coming for treatment?	45.3%(24)	47.2%(25)	7.5%(4)	0	0
Patient- therapist relationship	Do your patients perceive the treatment to have negative effect on him/her?	0	0	11.3%(6)	56.6%(30)	32.1%(17)
	Do your patients find the treatment duration to be longer than it should be	9.4%(5)	34.0%(18)	9.4%(5)	0	0
	Does the patient therapist relationship affect your treatment plans	15.1%(8)	52.8%(28)	5.7%(3)	0	0

Regarding the patient's compliance factors, a majority (30, 56%) of the respondents strongly agreed that patients attend fixed appointments, either weekly or for up twice or more than in a month. Majority (31, 58.5%) of the participants also agreed that patients occasionally forget to come on treatment schedule days. With respect to the distance related factors, a huge number of participants (31, 58.5%) agreed that distance of the clinic and absence of caregivers prevents patient's coming for treatment. From the participants' point of view; treatment (25, 47.2%) and transportation (29, 54.7%) costs prevents patients from coming to the clinic. Respondents also agreed (28, 52.8%) that their relationship with patients affect their treatment plans and disagreed (30, 56.6%) that their patients perceive the treatment have negative effect on them. 5 (9.4%) and 18 (34.0%) of the respondents respectively strongly agreed and agreed that patients find the treatment duration to be longer than it should be. Details are found in Table 2.

## Discussion

This study presents findings on the extent in which some factors influence patient's adherence to physiotherapy treatment, hence the development of treatment schedules by their physiotherapists. Participants' responses were obtained based on four broad factors which are likely to influence patients' adherence to treatment and as a result inform the therapist's decision on developing a treatment schedule for them. They agreed to varying extents that distance related factors and economic factors (transportation and cost of treatment) prevent patients from attending their treatment sessions.

### Compliance level factors

From a physiotherapy context, an adherent behavior constitutes attending clinical appointment, actively participating in rehabilitation activities during clinical appointments, carrying out home programs, avoiding contraindicated activities and using recommended protective or therapeutic device^13^. However, this study laid emphasis on an adherent behavior which constitutes attending clinical appointments. Forgetfulness has been reported as a major cause of non-compliance to clinic appointments^14^, ^15^, ^16^ and findings from this study shows that patients miss their appointment days due to forgetfulness consequently hindering the delivery of optimal care. Other factors which can lead to poor adherence to physiotherapy treatment includes, an inadequate knowledge of caregivers' on the role of physiotherapy in cerebral palsy management and how they perceive the relevance of and adherence to the treatment programs. If parents/caregivers attach a negligible importance to these factors, it could lead to forgetfulness and poor behavior towards treatment adherence. With respect to children with cerebral palsy, the onus of adherence to treatment lies with the caregivers or parents, highlighting the importance of factoring in patient's treatment schedules; an intervention focused on parents' education to improve their knowledge and ability to adhere to these treatment programs. Interventions such as peer support relationship can be effective in enabling patients adopt a positive attitude to treatment and adherence; mobile phone reminders/texts for the purpose of curbing forgetfulness thus boosting adherence. hese factors ought to be considered while developing treatment schedules for patients with cerebral palsy.

### Distance-related factor

Distance to clinic could also be a major reason why patients fail to come for treatment. Persons living with disabilities^17^ and cerebral palsy experience greater barriers to accessing health services when compared to their counterparts. Most health facilities in Nigeria are located in urban areas partly due to resource constraints hence warranting people living in rural areas to travel long distances in a bid to access health care. There is also an added burden of bad roads and poor road networks. Distance barriers specifically lead to rescheduling of treatment appointment, delay of treatment and missed treatment session. All these could culminate to poor adherence to treatment, management and thus, health outcomes. Distance as barrier to health services becomes much burdensome to patients with low income^18^ highlighting the relationship between socioeconomic and distance related factors of patient's adherence. Thus, governments should revamp road networks and open up health facilities at the rural areas to foster easier access and referral based on proximity issues. Clinicians on the other hand, should put this into consideration while developing treatment schedules for such patients and also discuss with the patients on probable ways of minimizing the chances of missed appointments.

### Economic status factor

Low economic status and lack of income are important risk factors of non-adherence to different treatments^19^,^20^. This is also evidenced in our findings as majority of participants strongly agreed that the cost of treatment influenced their patients from coming for treatment. These findings showed that healthcare expenditure comprising of cost of treatment and transportation could constitute a large portion of living expenses for patients with cerebral palsy. Cost and income are two interrelated factors. Healthcare cost is less burden when the patient has a relatively high income or health insurance. Specifically, a number of studies indicated that patients who had no insurance cover^21^, ^22^, ^23^ or who had low income 24, 25 may not likely comply to the treatment schedule days due to the cost for treatment. They are also likely to have a relatively worse self-reported health, suffer from more chronic conditions and have a lower life expectancy because of limited access to health care due to cost and coverage^26^. Noted in this study is the additional burden of transportation cost. Affordability and availability of transport to health facilities, coupled with prolonged travel time pose as a barrier to accessing health care^27^ and hence influences patient's adherence to treatment. Therefore, healthcare providers should be aware of patient's economic situation^28^ and also be at par with the active projects in place to reduce the socioeconomic and access to health care disparities in the country.

### Patient-Therapist relationship factors

It is quite satisfying that a good number of the respondents disagreed that their patients do perceive the treatment to have negative effect on them. Patient's perception of therapeutic benefits, patient's beliefs, motivation and attitude towards therapy were identified as factors to be included in influencing treatment compliance. Misconceptions or erroneous beliefs held by patients could contribute to poor compliance to appointment^12^. Specifically, patient's worries about the treatment, believing that the disease is controllable or attributing it to religious belief might add to the likelihood that they are unable to adhere to their treatment appointment. Negative attitude towards therapy (depression, anxiety, perceived feeling of exacerbation of symptoms, fears etc.) and their compliance to treatment schedules^29^, ^30^, ^22^, ^31^. Our findings also showed that parents agreed that their treatment duration were longer than it should be. When patients were worried that their duration of treatment is lengthy, they were likely to have poor compliance to the appointment days^28^,^32^. Proper scheduling strategies could be utilised to ensure that patient load per time doesn't exceed the staff strength of the paediatric unit. Failure to resolve this may lead to patients clustering and waiting for a longer period of time. All these aforementioned factors can be addressed by fostering a good patient-therapist relationship. Patient-therapist relationship is a strong factor which affects patients' compliance to treatment^29^, ^24^, ^35^, ^36^, ^37^. A healthy relationship is based on patient's trust in their clinicians especially with delivering positive benefits for patients with cerebral palsy. Studies have found that patient response to treatment is good when therapists are emotionally supportive, giving reassurance or respect, and treating patients as an equal partner^38^, ^39^. In this study, majority of the respondents strongly believed that patient-therapist relationship affects their treatment plans. However, focus should be further channeled in ensuring the development and maintenance of a good patient-therapist relationship. These findings demonstrate the need for cooperation between patients and clinician and the importance of good communication. Communication between parents and their therapists is pertinent because it will avail a conducive environment for educating them on the need to adopt and maintain a positive behavior to health and treatment. Poor communication with the healthcare providers was also likely to cause a negative effect on patient's level of compliance to treatment appointment^40^, ^28^. To build a good and healthy patient-therapist relationship, physiotherapist should have patients involved in designing their treatment goals and plan^37^,^41^ and give patients a detailed explanation about the condition and treatment^42^, ^31^.

There are a few limitations in this study. First, this study presents findings from a single tertiary hospital that serves as the major referral center in South-Eastern Nigeria. It does not represent the general population of physiotherapists in Nigeria, hence findings from this study should be interpreted with caution. Furthermore, this study focused on attending clinical appointments as an adherent behavior. However, a more holistic assessment of patient's adherence to physiotherapy treatment including actively participating in rehabilitation activities during clinical appointments, carrying out home programs, avoiding contraindicated activities and using recommended protective or therapeutic devices is recommended. Future research is also encouraged to explore other factors such as increased workload and specific goals of recovery as an indices for outcome of therapy which were not featured in this study.

## Conclusion

Findings from this study indicates that factors such as economic status of patients and relationship between patients and their therapists; with the distance to the clinic playing a key role; influence treatment adherence for patients with cerebral palsy. It is also pertinent that patients adopt a positive behavior towards health as it could influence the treatment plans of such patients. These factors should be therefore be considered while developing a comprehensive treatment schedule for patients with cerebral palsy. Finally, factors influencing patients with cerebral palsy adherence to their treatment schedules should be explored and assessed from the parent's/caregiver's viewpoint.
